# Supplementary material for: Strategies for achieving a healthy oral cholera vaccine market: Model-enabled scenario exploration of supply and demand dynamics
Source: PLoS Negl Trop Dis. 2026 Feb 26;20(2):e0013995. doi: 10.1371/journal.pntd.0013995 (PMC12959686; doi:10.1371/journal.pntd.0013995)
Supplement: S1 Appendix — Table B: Defining key KPIs based on stakeholder discussions. Table C: Tradeoffs across key KPIs for scenarios 4–7. Table D: Overview of model parameters – regulatory approval subsystem. Table E: Values for model parameters arrayed by product – regulatory approval subsystem (optimistic & pessimistic). Table F: Overview of model parameters – global OCV policy subsystem. Table G: Overview of model parameters – demand subsystem. Table H: Impact of health system strengthening and outbreak preparedness. Table I: Seasonal distribution of reactive orders per month. Table J: Country specific data. Table K: Overview of model parameters – product development subsystem. Table L: Values for model parameters arrayed by product. Table M: Overview of model parameters – production and supply subsystem. Table N: Overview of model parameters – order fulfilment subsystem. Table O: Overview of model parameters – climate and conflict subsystem. Fig A: Evolution of WHO recommendations and OCV supplier landscape, with a focus on key milestones around technology transfer and WHO pre-qualification. The mapping also includes publicly reported OCV development efforts. OCVs have only been recommended as part of broader cholera control strategies since 2010. Fig B: Comparison of daily simulated OCV inventory levels (grey) to weekly reported OCV inventory levels (green) reported by UNICEF (2021–2024) [58]. The target level for the emergency OCV stockpile (red) is 5 million doses during the period presented. Fig C: Ratio of annual doses delivered to reactive (red) versus preventive (blue) settings for scenario 1–4. Fig D: Annual doses requested across all demand settings, scenarios 1–4. Fig E: A) Total programmatic cost, including OCV procurement and operational support for campaigns, for the entire simulation (2013–2035) for scenarios 1–4. B) Costs during the GAVI 5.0 funding period (2021–2025). C) Costs during the GAVI 6.0 funding period (2026–2030). D) Costs during the GAVI 7.0 funding period ( [file pntd.0013995.s001.docx]

**Appendix to *Strategies for achieving a healthy OCV market***

**A. Stakeholder engagement and key performance indicators**

**Table A**: Interviewees categorized by organization type.

| **Organization type** | **Number of interviews** |
| --- | --- |
| National research center | 3 |
| Regional or international non-profit organization | 7 |
| Multilateral agency | 11 |
| Industry or consulting firm | 10 |
| Foundation | 5 |
| *Total* | **36** |

**Table B**: Defining key KPIs based on stakeholder discussions.

| **KPI** | **Definition** | **Relevance** |
| --- | --- | --- |
| **OCV doses requested** | Cumulative and annual demand for OCV across both preventive and reactive settings | Doses **countries** need to meet cholera-related immunization efforts, as part of broader multi-sectoral activities |
| **OCV doses produced** | Cumulative and annual doses produced across all WHO pre-qualified suppliers in the market | Doses available from **suppliers** over time |
| **Countries adopting preventive OCV vaccination** | Number of countries adopting multi-year preventive programs and timing of applications for GAVI support | Indication for how many **countries** are transitioning towards preventive-focused vaccination strategies |
| **Unfulfilled decision letters** | Doses requested and approved (decision letter has been sent), but order has not yet been made | Signals potential delays in delivering doses to countries |
| **Number of suppliers** | Number of WHO prequalified suppliers in the market | Multiple **suppliers** reduces supply risks and increased overall supply capacity of the market |
| **Doses delivered** | Number of doses that have been delivered to countries and for what purpose | The ratio of doses delivered to reactive versus preventive settings points to **country** transition from crisis response to preventive efforts for cholera control, thus more predictable demand for suppliers |
| **Cost of OCV procurement and program support** | Cumulative and annual costs for OCV programmatic efforts, including procurement and implementation support | Indication for programmatic feasibility under different supply and demand scenarios, particularly relevant for **procurement agencies** and **funders** |
| **Likelihood of allowing 2-dose reactive vaccination** | Fraction of simulation time, between 2025 and 2035, for which 2-dose reactive vaccination is allowed, based on a pre-defined policy | Overall measure of how often 2-dose reactive vaccination is possible in reactive settings, in line with WHO SAGE recommendations |
| **Likelihood of allowing preventive vaccination** | Fraction of simulation time, between 2025 and 2035, for which preventive vaccination is allowed, based on a pre-defined policy | Overall measure of how often preventive vaccination is possible in endemic countries, in line with WHO SAGE recommendations |
| **Stockpile readiness** | Fraction of simulation time, between 2025 and 2035, for which the inventory level is above the stockpile’s target emergency reserve level | Overall measure of supply security |

**B. Supplier landscape**

Few suppliers have attained the required WHO prequalification (PQ) for UNICEF procurement. A historical overview of the OCV supply landscape is presented in **Figure A**. Products such as Cholvax (Incepta, Bangladesh) and mORCVAX (VaBiotech, Vietnam) have been used for years, but are not licensed by WHO-listed agencies as required for WHO PQ. Since Shantha Biotechnics (India) left the market in 2023, EuBiologics (South Korea) remains the only supplier eligible to replenish the global stockpile. To increase global production capacity, several technology transfer efforts have been initiated by the International Vaccine Institute, while other products progress through the development pipeline. These insights were important to make assumptions around the expected entry of new suppliers during the simulation period.


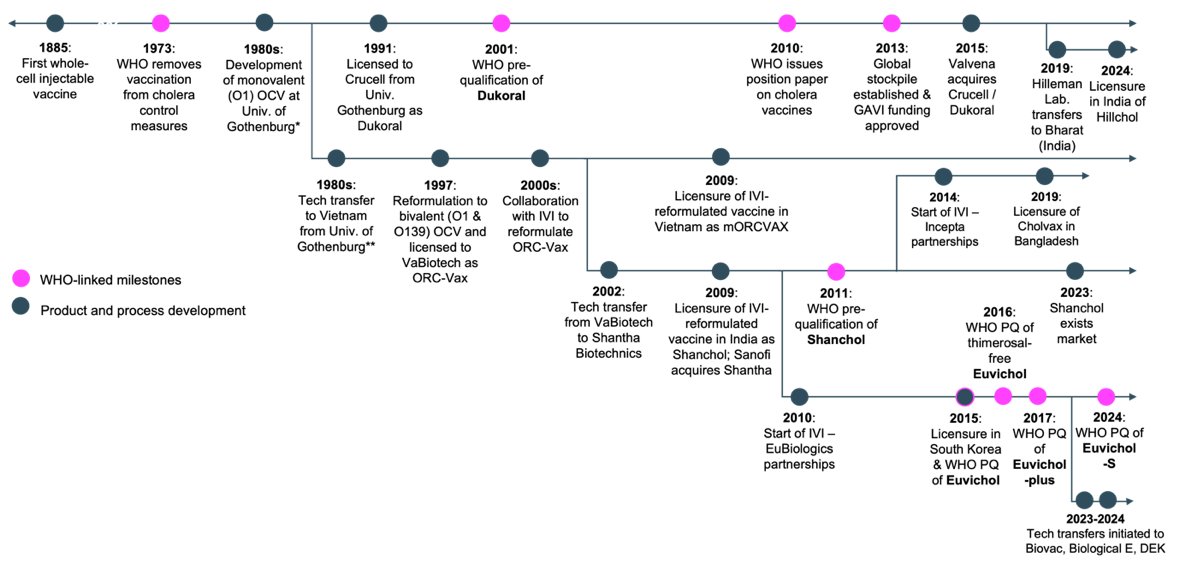


**Fig A**: Evolution of WHO recommendations and OCV supplier landscape, with a focus on key milestones around technology transfer and WHO pre-qualification. The mapping also includes publicly reported OCV development efforts. OCVs have only been recommended as part of broader cholera control strategies since 2010.

**C. Model validation**

A unique characteristic of system dynamics is the ability to capture changes in system behavior by reflecting unique stakeholder perspectives, processes, and decision points. Extensive validation interviews led to iterative model refinement to structures (e.g., feedback loops), ensuring model behavior aligns with that expected in the real-world. Stakeholders expressed that the model conceptually and structurally captures the main issues currently seen in the OCV market. Furthermore, the model replicates the behavior of UNICEF-reported inventory levels of the global stockpile (**Figure B**) between 2022 and 2024. The values for the root mean squared error (RMSE), R^2^, and Theil inequality (U) are statistical measures for model validity. Specifically, the RMSE normalized by the sample range is 0.23, while the R^2^ is 0.4, and U is 0.25. Theil’s inequality can be further decomposed using component analysis: unequal means (U_m_), unequal variances (U_s_), and imperfect correlation (U_c_) [1]. For the data shown, U_m_ = 0.14, U_s_ = 0.01, and U_c_ = 0.85. The mean (U_m_) and variance (U_s_) shows errors are unsystematic, meaning the model effectively captures variability in observed data with little bias. Most errors come from misalignments in the timing of changes between the actual and simulated stockpile levels, with very high and frequent variability.

Although the model behavior is appropriate, several reasons can explain deviations in the timing of simulated dynamics. First, the stockpile level emerges from complex, simultaneous supply and demand processes. Second, errors can be traced to the production schedules of suppliers and timing of production processes. Since the model aggregates multiple processes into a few production flows, it may not account for some delays seen in the real-world. Third, purchase orders are fulfilled between weekly UNICEF reports on supply availability, though not documented publicly. Looking only at data from 2024 is informative since supply is most predictable due to full facility utilization. In this period, the normalized RMSE is 14%, R^2^ is 0.82, and U is 0.15 (Um = 0.06, Us = 0.00, and Uc = 0.94). This means that the model fit in 2024 is better than for previous years, accounting for more of the variance observed in real-world data. In the future, preventive vaccination will lead to a rapid increase in demand, with the capacity of new suppliers expected to be fully utilized. Therefore, future behavior will resemble the supply constrained environment seen in 2024.

**Fig B**: Comparison of daily simulated OCV inventory levels (grey) to weekly reported OCV inventory levels (green) reported by UNICEF (2021-2024)[2]. The target level for the emergency OCV stockpile (red) is 5 million doses during the period presented.

**D. Supplemental results**

**Fig C**: Ratio of annual doses delivered to reactive (red) versus preventive (blue) settings for scenario 1-4.

**Fig D**: Annual doses requested across all demand settings, scenarios 1-4.

**Fig E**: A) Total programmatic cost, including OCV procurement and operational support for campaigns, for the entire simulation (2013-2035) for scenarios 1-4. B) Costs during the GAVI 5.0 funding period (2021-2025). C) Costs during the GAVI 6.0 funding period (2026-2030). D) Costs during the GAVI 7.0 funding period (2031-2035).

**Table C**: Tradeoffs across key KPIs for scenarios 4-7.

| **Scenario** | **^a^ T_r_**  (%) | **^b^ T_p_**  (%) | **^c^ T_s_**  (%) | **^d^ R_r_**  (million) | **^e^ R_p_**  (million) |
| --- | --- | --- | --- | --- | --- |
| 4 | n/a | 27.3 | 73.5 | 423 | 1165 |
| 5 |  | 24.3 | 73.8 | 394 | 1193 |
| 6 |  | 27.6 | 82.1 | 452 | 941 |
| 7 |  | 16.9 | 70.5 | 395 | 1312 |
| 8 |  | 17.1 | 71.0 | 412 | 1287 |
| 9 |  | 26.1 | 77.3 | 435 | 1062 |

*Note:* ^a^**T_r_**: Fraction of forecasted simulation time (2025-2035) for which 2-dose reactive vaccination is allowed; ^b^**T_p_**: Fraction of forecasted simulation time (2025-2035) for which preventive vaccination is allowed; ^c^**T_s_**: Fraction of simulation time (2025-2035) for which the inventory level is above the stockpile’s target emergency reserve level; ^d^**R_r_**: Doses requested in reactive settings; ^e^**R_p_**: Doses requested in preventive settings.

**Fig F**: Cumulative doses requested for scenarios 10-14, compared to scenario 4 (no budget constraint), across reactive (A), preventive (B), and all (C) settings.

**Fig G**: Approved requests that have not been delivered for scenarios 10-14 compared to scenario 4 (no budget constraint).

**Fig H**: Approved request that have not been delivered for scenarios 14-20, considering different polices for resuming preventive vaccination and two-dose reactive vaccination.

**D. Baseline parameter values and assumptions**

Parameters that are dimensionless have units *dmnl.* Parameters that directly influence behavior in multiple subsystems are only documented once, in the originating subsystem. Baseline values reported for static parameters refer to assumptions in scenario 1. If the parameters are dynamic, the table reports the initial value at the start of the simulation.

*C.1 Regulatory approval*

Regulatory authorization, at both a global and national level, is necessary for access to safe and effective vaccines. Capturing regulatory delays faced by suppliers is important as it can limit the availability of vaccines. Since OCV is largely purchased by UNICEF through the public market, suppliers are required to attain WHO prequalification (PQ) for their products. Prior to WHO PQ, vaccines need market authorization from at least one stringent national regulatory authority (NRA), those considered to be highly function (Maturity Level 3 or 4) based on WHO’s Global Benchmarking Tool.

Due to the time-critical nature of reactive demand, countries requesting vaccines from the global OCV stockpile do not need to grant market authorization (if it hasn’t previously been done). For preventive campaigns, however, country-level market authorization is needed to benefit from multi-year programmatic support from GAVI. Regulatory approval by individual NRAs following WHO PQ is not considered in the model.

At the start of the simulation, when the OCV stockpile is established, Dukoral (2001) and Shanchol (2011) have already received WHO PQ. However, only Shanchol is procured by UNICEF given its preferred product characteristics. Additionally, several non-WHO PQ vaccines have been approved by NRAs (e.g., mORCV-VAX in Vietnam in 2009). During the simulation period, EuBiologics emerges as a new supplier, with three WHO PQ products: Euvichol (2015), Euvichol-Plus (2017), and Euvichol-S (2024). Due to continued supply gaps, several development and technology transfer efforts lead to new suppliers submitting dossiers for WHO PQ (these are be modeled in a supplier-agnostic manner). The model is not designed to predict which supplier will first attain WHO PQ and be eligible for public procurement. Rather, it captures how each supplier’s time to entry into market and production capacity impact dynamics of OCV supply and demand.

Model assumptions are valid for all OCV products (∀*p*) in the set of products P = {Shanchol, Euvichol, Euvichol-Plus, Euvichol-S, Product 1, Product 2, Product 3}

**Table D***:* Overview of model parameters – regulatory approval subsystem.

| **Parameter** | **Type** | **Units** | **Array dimensions** | **Baseline value** |
| --- | --- | --- | --- | --- |
| Accelerated review status by SNRA per product^1^ | switch | dmnl | P | 0  ∀*p* ∈ P |
| Effect of SNRA review status  on review time | static | dmnl | n/a | 0.5 |
| Expected SNRA  review time per product | static | days | P | see table 5 |
| Realized time  to SNRA approval per product | static | days | P | n/a |
| Time of dossier  submission to SNRA per product^2^ | static | days | P | see table 5 |
| Time from SRNA approval to  WHO PQ dossier submission per product^3^ | static | days | P | see table 5 |
| Accelerated review status by WHO PQ per product^4^ | switch | dmnl | P | see table 5 |
| Effect of accelerated WHO PQ review status on review time | static | dmnl | n/a | 0.5 |
| Expected WHO PQ  review time per product | static | days | P | see table 5 |
| Realized time  to WHO PQ approval per product | static | days | P | n/a |
| Attaining SNRA  and WHO PQ  approval per product | dynamic | dmnl | P | n/a |
| Products with  WHO PQ status | static | dmnl | n/a | 0 |
| New suppliers entering market^5^ | switch | dmnl | n/a | 0 |
| Scenario for new  suppliers to enter market^6^ | static | dmnl | n/a | 0 |
| Risk level of change^7^ | static | dmnl | n/a | 0 |

^1^ Refers to the SRNA in the country in which a product is produced. Serves as a logical switch in the model: 0 – normal review timeline, 1 – accelerated review timeline.

^2^ Time elapsed relative to the start of the simulation.

^3^ Can be negative if the WHO PQ dossier was submitted prior to a decision by the SRNA. These reviews are sometimes done in parallel to reduce overall delays.

^4^ Refers to the WHO pre-qualification program. Serves as a logical switch in the model: 0 – normal review timeline, 1 – accelerated review timeline.

^5^ Refers to new OCV suppliers seeking WHO PQ. Serves as a logical switch in the model: 0 – no new suppliers during simulation, 1 – three new suppliers between 2025 and 2035.

^6^ Defining scenario for market entry of new suppliers: 0 – no new OCVs with WHO PQ, 1 – optimistic scenarios, 2 – pessimistic scenario.

^7^ Refers to changes to dossiers following WHO PQ; depending on modifications made, review times can range between 30 days and 6 months.

**Table E***:* Values for model parameters arrayed by product – regulatory approval subsystem (**optimistic** & **pessimistic**).

| **Parameter** | **Units** | **Euvichol** | **Euvichol-Plus** | **Euvichol-S** | **Product 1** | **Product 2** | **Product 3** |
| --- | --- | --- | --- | --- | --- | --- | --- |
| Time of dossier  submission to SNRA per product | date | Sept. 5, 2014 | Sept. 30, 2016 | Mar. 15, 2023 | Jan. 1, 2024 | Jan. 1, 2026 | Jan. 1, 2027 |
| Expected SNRA  review time per product | days | 145 | 180 | 279 | 244  244 | 243  365 | 243  366 |
| Time from SRNA approval to  WHO PQ dossier submission per product | days | -9 | 0 | -157 | 30  122 | 30  31 | 30  31 |
| Accelerated review status by WHO PQ per product | dmnl | 0 | 1 | 0 | 0  0 | 0  0 | 0  0 |
| Expected WHO PQ  review time per product | days | 330 | 270 | 270 | 273  365 | 273  334 | 274  334 |

*F.2 Global OCV policy*

Recommendations made by WHO on the use of OCVs with WHO PQ are informed by the Strategic Advisory Group of Experts (SAGE). Often, these recommendations serve as a blueprint for guidance provided by regional and national immunization technical advisory groups. Additionally, the International Coordinating Group on Vaccine Provision (ICG) and Global Task Force on Cholera Control (GTFCC), given their role around governance of the emergency OCV stockpile and allocation of doses for preventive campaigns, respectively, contribute to when and how demand is met. The same recommendations and decisions are made for all OCV products, regardless of the supplier. For this subsystem, data is based on publicly available and historic decisions. Additionally, data is used from GAVI’s Vaccine Funding Guidelines [3], providing information on what programs GAVI supports and considerations for applying for new vaccine support.

The focus of this subsystems is on a few key decisions:

- Target emergency reserve in global stockpile
  - Initially set at 2 million doses (2013), then increased to 3 million doses in 2019 and 5 million doses in 2022.
  - For all scenarios presented, the target reserve is kept at 5 million doses. However, this value can also be user-defined or defined endogenously each year based on various parameters, including the total production capacity, number of suppliers in the market, and demand in reactive settings.
  - Performance: stockpile readiness (see Table S2)
- Guidance around the use of vaccines in preventive settings
  - Dosing schedule in normal times
  - Decision to resume vaccination in preventive settings, as of 2025, which have been formally suspended since October 2022 due to supply scarcity
  - Decision on revaccination following initial preventive vaccination program
  - Duration of preventive program and frequency of campaigns
  - Performance: likelihood of allowing preventive vaccination (see Table S2)
- Guidance around the use of vaccines in reactive settings
  - Dosing schedule in normal times
  - Decision to resume two-dose vaccination in reactive settings, as of 2025, which has been formally suspended since October 2022 due to supply scarcity
  - Performance: likelihood of allowing 2-dose reactive vaccination (see Table S2)

**Table F***:* Overview of model parameters – global OCV policy subsystem.

| **Parameter** | **Type** | **Units** | **Array dimensions** | **Baseline value** |
| --- | --- | --- | --- | --- |
| Dynamic stockpile policy switch^8^ | switch | dmnl | n/a | 0 |
| User-defined target stockpile level | static | doses | n/a | 5 million |
| Target stockpile level | dynamic | doses | n/a | 5 million |
| Resuming preventive vaccination switch^9^ | switch | dmnl | n/a | 0 |
| Resuming two dose vaccination in outbreak settings vaccination switch^10^ | switch | dmnl | n/a | 0 |
| Date to resume preventive vaccination indefinitely | static | date | n/a | n/a |
| Date to resume reactive boosters indefinitely | static | date | n/a | n/a |
| Decision to resume preventive vaccination campaigns | dynamic | dmnl | n/a | 0 |
| Decision to resume two dose vaccination in outbreak settings | dynamic | dmnl | n/a | 0 |
| Interval between initial preventive program and follow-up program | static | days | n/a | 1080 |
| Interval time between campaigns in preventive program | static | days | n/a | 360 |
| Moving average supply threshold to resume preventive vaccination | dynamic | dmnl | n/a | 0 |
| Moving average supply threshold to resume reactive boosters | dynamic | dmnl | n/a | 0 |
| Number of phases planned in preventive program | static | dmnl | n/a | 3 |
| Recommendation for revaccination following initial preventive campaign^11^ | switch | dmnl | n/a | 0 |
| Recommended dosing schedule for preventive vaccination | static | doses / people | n/a | 2 |
| Recommended dosing schedule for reactive campaigns | static | doses / people | n/a | 1 |
| Supply threshold to resume preventive vaccination | dynamic | doses | n/a | 15 million |
| Supply threshold to resume reactive boosters | dynamic | doses | n/a | 5 million |
| DP WIP threshold to resume preventive vaccination | dynamic | doses | n/a | 30 million |
| total WIP threshold to resume preventive vaccination | dynamic | doses | n/a | 60 million |

^8^ Annual decision to review the target stockpile level: 0 – off, 1 – on.

^9^ Decision to resume preventive vaccination: 0 – off, 1 – on.

^10^ Decision to resume two-dose reactive vaccination: 0 – off, 1 – on.

^11^ Decision to revaccinate following initial preventive campaigns: 0 – off, 1 – on.

*F.3 OCV demand & orders*

In endemic countries outbreaks are defined as an unexpected increase of suspected cases over 2 consecutive weeks, of which some are laboratory confirmed. For non-endemic countries, an outbreak is defined as the occurrence of at least one confirmed case of cholera and evidence of local transmission. The use of vaccines following humanitarian emergencies, when infrastructure may be damaged and routine services are unavailable, is meant to reduce the risk and spread of cholera transmission. Definitions are in line with those from the GTFCC.

Data was collected from ICG’s cholera vaccine dashboard^^[[1]](#footnote-2)^^, presenting all country requests for use of OCV in outbreak and humanitarian settings. Data provided include: date of ICG decision, status (approved, partially approved, not approved, or cancelled by requestor), context (e.g., outbreak response, humanitarian crisis), number of doses requested, number of doses approved, and number of doses shipped. Data was cross-checked with the GTFCC’s OCV dashboard^^[[2]](#footnote-3)^^, developed by Epicentre (Médecins Sans Frontières), as it has more granular data and also reports requests for preventive use. Discrepancies were resolved based on the best available information across both sources. Data covers the period between January 2013 and December 2024. Data was processed in Excel and uploaded to Stella.

In some cases, multiple submissions were made under the same request, for example to adjust the vaccination strategy as an outbreak evolved. These were merged, leading to 190 ICG requests. When requests are approved by the ICG, but 0 doses are shipped, we assume orders have been cancelled. Requests can also be cancelled after submission, leading to no doses being approved nor shipped. To assess seasonality of ICG requests, data was pooled into different months. As shown in **Figure S10**, periods with the most reactive demand, as a fraction of all historic ICG requests, were November (13.1%), June (12.2%), October (11.1%), March (10.3%), and September (10.0%). Additionally, as shown in **Figure S11**, several countries account for a large proportion of historic ICG requests: Ethiopia (16%), Sudan (9%), Nigeria (8%), Bangladesh (7%), DRC (7%), and Cameroon (7%). Between 2013-2024, two-thirds of reactive demand occurred in the last three years (2022, 2023, and 2024).

**Fig I**: Seasonality of historic ICG requests, reported monthly and for each year between 2013-2024 (left axis, colored points) and the average proportion of requests over all years happening in each month (right axis, red line).

**Fig J**: Geographic distribution of historic ICG requests reported by country, cumulative between 2013-2024.

Based on 153 ICG requests with available data, the median time from request to decision was 5 days, with an interquartile range of (3,10). Based on 126 ICG requests with available data, the median time from decision to first shipment was 14 days, with an interquartile range of (10,20). Delays reviewing requests and shipping vaccines may be due to the need for additional data from countries, supply scarcity, or logistic barriers around campaigns implementation.

Future OCV demand for reactive use is calculated annually based on a year-to-year change in the expected demand. The timing of requests considers the seasonal distribution of historic requests, inter-arrival times between each request, and the impact of preventive vaccination. As of 2022, due to supply scarcity, 2-dose vaccination in reactive settings has been suspended to allow for greater coverage with a 1-dose strategy. Model assumptions are valid for all month (∀*m*) in the set of months M = {January,…,December} and years (∀*y*) in the set of years Y = {2025,…,2035}.

**Fig K**: Model structure of key stocks and flows – reactive demand.

A country is considered cholera endemic if it has an area (state, district, or smaller locality) where confirmed cases, resulting from local transmission, have been detected in the last 3 years. Given sustained (year-round) transmission or recurring cases, these regions are eligible for phased multi-year preventive vaccination campaigns and are called Priority Areas for Multisectoral Interventions (PAMIs). These PAMIs or hotspots allow for more targeted campaigns in geographically limited areas (e.g.. city, health district catchment area) where environmental, cultural and/or socioeconomic conditions facilitate transmission and where cholera persists or re-appears regularly.

Future preventive OCV demand is modeled based on a methodology defined by GAVI to generate strategic demand scenarios for OCV. It considers factors such as the rate of countries adopting preventive OCV vaccination and applying for programmatic support, changing at-risk population based on investments in improved WASH, target coverage, dosing schedule, time between campaigns, and GAVI eligibility. It was most recently used to estimate OCV demand between 2023 and 2032 as part of GAVI’s Market Shaping Roadmap for OCV. Importantly, countries need to show targeted OCV campaigns are complemented with multi-sectoral activities (e.g., investment in WASH infrastructure, surveillance, social mobilization). Since preventive vaccination has been paused in 2022, several assumptions have been adapted, such as the time when countries will apply for funding to support multi-year preventive campaigns. Model assumptions are valid for all countries (∀*c*) in the set of countries C = {Afghanistan,…,Dominican Republic} and years (∀*y*) in the set of months Y = {2025,…,2035}

**Fig L**: Model structure of key stocks and flows – preventive demand.

**Table G***:* Overview of model parameters – demand subsystem.

| **Parameter** | **Type** | **Units** | **Array dimensions** | **Baseline value** |
| --- | --- | --- | --- | --- |
| Reactive demand switch^12^ | switch | dmnl | n/a | 1 |
| Calculated annual change in reactive demand | dynamic | dmnl | n/a | n/a |
| Baseline annual  change in reactive demand | static | dmnl | n/a | 0 |
| Health system strengthening and outbreak preparedness switch^13^ | switch | dmnl | n/a | 0 |
| Impact of health system strengthening and outbreak preparedness | static | dmnl | n/a | see table S8 |
| Reactive requests (total over all countries) in 2024 annual | static | doses | n/a | 61,2654,64 |
| Seasonal distribution of reactive orders per month | static | dmnl | n/a | see table S9 |
| Date of decision from ICG per month per year | static | dmnl | n/a | First day of each month |
| Interarrival time of reactive orders | static | days | n/a | 7 |
| Geographic distribution of reactive orders per country | static | doses | C | see table S10 |
| Country ordering behavior switch^14^ | static | dmnl | n/a | 0 |
| Baseline effect of supply scarcity on reactive order size^15^ | static | dmnl | n/a | 65% |
| Baseline population at risk of cholera per country | static | people | C | see table S10 |
| Time for preventive campaigns to impact reactive demand | static | days | n/a | 365 |
| WASH switch^16^ | switch | dmnl | n/a | 0 |
| Start year for WASH intervention | static | dmnl | n/a | 2028 |
| End of year WASH intervention | static | dmnl | n/a | 2035 |
| Effect of WASH on size of at-risk population | static | dmnl | n/a | 50% |
| Target coverage for preventive vaccination^17^ | static | dmnl | n/a | 50% |
| Rapid diagnostics switch^18^ | static | dmnl | n/a | 0 |
| Effect of diagnostics on target population for preventive vaccination^19^ | static | dmnl | n/a | 75% |
| GAVI eligibility per country^20^ | static | dmnl | C | see table S10 |
| GAVI graduation switch^21^ | static | dmnl | n/a | 0 |
| Rate of adoption of preventive vaccination per country | static | dmnl | C | see table S10 |
| Population dynamics switch^22^ | switch | dmnl | n/a | 0 |
| Effect of demographic changes on at-risk population per country | static | dmnl | n/a | see table S10 |
| Size of at risk population per country | dynamic | people | C | n/a |
| Minimum time to submit application | static | days | n/a | 120 |
| Mean time to submit application | static | days | n/a | 270 |
| Maximum time to submit application | static | days | n/a | 360 |
| Time when at risk population is  estimated^23^ | stochastic | days | C | n/a |
| Minimum time for GAVI to review application | static | days | n/a | 60 |
| Mean time for GAVI to review application | static | days | n/a | 120 |
| Maximum time for GAVI to review application | static | days | n/a | 270 |
| Time for GAVI to review application | stochastic | days | n/a | n/a |
| Baseline effect of pending decision letters on adoption of preventive vaccination^24^ | static | days | n/a | 50% |

^12^ Consider vaccination in reactive settings: 0 – off, 1 – on.

^13^ Consider effect of health system strengthening and outbreak prevention: 0 – off, 1 – on.

^14^ Consider effect of order behavior in both reactive and preventive settings: 0 – off, 1 – on.

^15^ Assume that when the available supply inventory is below the target emergency reserve for the global OCV stockpile, reduce order size by 35% compared to a setting with no supply constraint.

^16^ Consider effect of large infrastructure investments in water, sanitation, and hygiene: 0 – off, 1 – on.

^17^ Assume the at-risk population is homogenously spread across each country and that only 50% of districts are above the incidence threshold for preventive vaccination. Additionally, assume 100% coverage within each district targeted for preventive vaccination.

^18^ Consider effect of rapid diagnostic testing: 0 – off, 1 – on.

^19^ Assume 16 countries have adopted rapid diagnostic testing in 2025, leading to a 25% reduction in the size of the target population for preventive vaccination per country.

^20^ Based on GAVI eligibility status in 2024.

^21^ Consider effect of GAVI graduation: 0 – off, 1 – on.

^22^ Consider effect of population dynamics on the size of the at-risk population, also accounting for access to basic clean water and sanitation (different from WASH infrastructure): 0 – off, 1 – on.

^23^ Calculated stochastically based on the time when countries apply for funding to support preventive vaccination.

^24^ Assume that if a country has not yet adopted preventive vaccination and unfulfilled decision letters are above 150 million doses, then they will submit applications for funding with a target population 50% below the baseline with no supply constraint.

**Table H**: Impact of health system strengthening and outbreak preparedness.

| **From (year)** | **To (year)** | **% change in annual reactive demand across all countries** |
| --- | --- | --- |
| 2024 | 2025 | 2.5 |
| 2025 | 2026 | 5 |
| 2026 | 2027 | 7.5 |
| 2027 | 2028 | 10 |
| 2028 | 2029 | 10 |
| 2029 | 2030 | 10 |
| 2030 | 2031 | 7.5 |
| 2031 | 2032 | 5 |
| 2032 | 2033 | 2.5 |
| 2033 | 2034 | 0 |
| 2034 | 2035 | 0 |

**Table I**: Seasonal distribution of reactive orders per month.

| **Month** | **% of yearly orders** |
| --- | --- |
| January | 7.89 |
| February | 3.03 |
| March | 10.32 |
| April | 6.98 |
| May | 5.27 |
| June | 12.20 |
| July | 9.61 |
| August | 4.26 |
| September | 9.97 |
| October | 11.13 |
| November | 13.09 |
| December | 6.25 |

**Table J**: Country specific data.

| **Country** | **Baseline population**  **at risk of cholera** | **Gavi eligibility** | **Rate of adopting preventive vaccination**^25^  **(fast // slow)** | **Geographic distribution of reactive demand (2013-2024)** | **Effect of demographic changes on at-risk population**^26^ |
| --- | --- | --- | --- | --- | --- |
| Afghanistan | 17890622 | 1 | 0 // 0 | 0 | 0.01 |
| Angola | 8210632 | 0 | 0 // 0 | 0 | 0.01 |
| Bangladesh | 66495209 | 1 | 1 // 1 | 7.29 % | -0.01 |
| Benin | 8273524 | 1 | 6 // 9 | 0 | 0.02 |
| Burkina Faso | 12898436 | 1 | 0 // 0 | 0 | 0.02 |
| Burundi | 4985687 | 1 | 6 // 8 | 0 | 0.02 |
| Cameroon | 10518415 | 1 | 2 // 2 | 7.02 % | 0.02 |
| Chad | 10197079 | 1 | 0 // 0 | 0.28 % | 0.03 |
| Congo DR | 47265282 | 1 | 1 // 1 | 7.33 % | 0.02 |
| Congo Republic | 3371606 | 1 | 0 // 0 | 0 | 0.03 |
| Cote d'Ivoire | 14422207 | 1 | 0 // 0 | 0 | 0.02 |
| Djibouti | 417018 | 1 | 0 // 0 | 0 | 0.01 |
| Eritrea | 5454101 | 1 | 0 // 0 | 0 | 0.02 |
| Ethiopia | 68805272 | 1 | 3 // 4 | 16.02 % | 0.01 |
| Ghana | 20866095 | 1 | 4 // 6 | 0.45 % | 0.01 |
| Guinea | 8918347 | 1 | 0 // 0 | 0 | 0.02 |
| Guinea-Bissau | 1269299 | 1 | 0 // 0 | 0 | 0.02 |
| Haiti | 8214012 | 1 | 0 // 0 | 1.24 % | 0.01 |
| India | 411700175 | 0 | 0 // 0 | 0 | 0 |
| Kenya | 27818252 | 1 | 4 // 5 | 3.36 % | 0.02 |
| Liberia | 3245552 | 1 | 0 // 0 | 0 | 0.02 |
| Malawi | 7356710 | 1 | 2 // 2 | 5.96 % | 0.02 |
| Mali | 10909050 | 1 | 0 // 0 | 0 | 0.03 |
| Mozambique | 19653157 | 1 | 1 // 1 | 5.51 % | 0.02 |
| Myanmar | n/a | 1 | 0 // 0 | 1.61 % | 0 |
| Nepal | 18523751 | 1 | 5 // 7 | 0.69 % | -0.01 |
| Niger | 14463309 | 1 | 6 // 10 | 3.22 % | 0.04 |
| Nigeria | 110198368 | 1 | 3 // 3 | 7.79 % | 0.03 |
| Pakistan | n/a | 1 | 6 // 8 | 3.41 % | 0 |
| PNG | 3772420 | 1 | 0 // 0 | 0 | 0.02 |
| Sierra Leone | 5004219 | 1 | 5 // 7 | 0.59 % | 0.02 |
| Somalia | 7419853 | 1 | 5 // 6 | 3.45 % | 0.03 |
| Sudan | 26382481 | 1 | 4 // 5 | 8.85 % | 0.02 |
| South Sudan | 7356287 | 1 | 2 // 3 | 2.29 % | 0.02 |
| Tanzania | 40475997 | 1 | 5 // 6 | 0 | 0.03 |
| Togo | 6216271 | 1 | 6 // 9 | 0 | 0.02 |
| Uganda | 22431561 | 1 | 5 // 7 | 0.09 % | 0.03 |
| Yemen | 10698614 | 1 | 3 // 4 | 2.48 % | -0.01 |
| Zambia | 6872832 | 1 | 3 // 4 | 3.27 % | 0.03 |
| Zimbabwe | 7846187 | 1 | 4 // 5 | 3.58 % | 0.02 |
| Namibia | 1481698 | 0 | 0 // 0 | 0 | 0.01 |
| Iran | n/a | 0 | 0 // 0 | 0 | 0 |
| Iraq | n/a | 0 | 0 // 0 | 0 | 0 |
| Thailand | n/a | 0 | 0 // 0 | 0 | 0 |
| Philippines | 24295524 | 0 | 0 // 0 | 0 | 0 |
| Malaysia | n/a | 0 | 0 // 0 | 0 | 0 |
| Dominican Republic | 1702855 | 0 | 0 // 0 | 0.05 % | 0.01 |

^25^ Two different settings are considered for the rate of adoption of preventive vaccination across countries. For fast adoption, 3-5 countries apply for funding each year between 2024-2030. For slow adoption, 1-3 countries apply for funding each year between 2024-2033. The number refers to the relative year from 2024 when countries are expected to apply for funding for preventive vaccination.

^26^ Annual change in the size of the population at risk of cholera, accounting for sanitation coverage, based on Kim et al. 2016 [4]. Specifically, the study calculates the size of the population across countries based on UN projected population and data from the WHO/UNICEF Joint Monitoring Programme.

*F.4 OCV product development*

Product properties can have a significant impact on OCV supply and demand dynamics. For example, the number of serotype strains in a vaccine’s formulation will have a direct impact on the production process time and throughput. As a result, it may be necessary to scale-up production to meet annual demand. Additionally, clinical properties such as vaccine efficacy (measured in clinical trial settings) and effectiveness (measured in real-world settings) are important drivers of vaccine-induced immune protection, as well as risks of future transmission, especially during outbreaks. In this model, the impact of preventive vaccination on future outbreak risks, measured by reactive OCV demand, uses high-level heuristics not epidemiological modeling. Although other product properties exist, only the most relevant ones within the scope of the model are defined.

**Table K**: Overview of model parameters – product development subsystem.

| **Parameter** | **Type** | **Units** | **Array dimensions** | **Equation** | **Baseline value** |
| --- | --- | --- | --- | --- | --- |
| Duration of vaccine  induced protection  after full vaccination | static | years | P | n/a | see table S12 |
| Number of strains | static | dmnl | P | n/a |  |
| DS shelf life | static | years | P | n/a |  |
| DP shelf life | static | years | P | n/a |  |
| Vaccine efficacy | static | dmnl | P | n/a |  |
| Relative effectiveness  to vaccine efficacy | static | dmnl | P | n/a |  |

**Table L**: Values for model parameters arrayed by product.

| **Product** | **Duration of vaccine**  **induced protection**  **after full vaccination** | **Number of strains** | **DS shelf life** | **DP shelf life** | **Vaccine efficacy** | **Relative effectiveness**  **to vaccine efficacy** |
| --- | --- | --- | --- | --- | --- | --- |
| **Shanchol** | 3 | 5 | 4 | 2 | 60% | 1.2 |
| **Euvichol** |  | 5 |  |  |  |  |
| **Euvichol-Plus** |  | 5 |  |  |  |  |
| **Euvichol-S** |  | 2 |  |  |  |  |
| **Product 1** |  | 2 |  |  |  |  |
| **Product 2** |  | 2 |  |  |  |  |
| **Product 3** |  | 2 |  |  |  |  |

*F.5 OCV production & supply*

This subsystem models the production of bulk drug substance (DS) and finished drug product (DP) of OCV, at an aggregate level. Given the difficulty of obtaining precise data on company-specific production processes, the values presented are approximations used to generate the expected production throughput and stockpile dynamics over time. It also includes the delivery of doses from the supplier warehouse(s) to countries, based on pending purchase orders. Model assumptions are valid for all OCV products (∀*p*) in the set of products P = {Shanchol, Euvichol, Euvichol-Plus, Euvichol-S, Product 1, Product 2, Product 3}.

**Fig M**: Model structure of key stocks and flows – production and supply.

**Table M:** Overview of model parameters – production and supply subsystem.

| **Parameter** | **Type** | **Units** | **Array dimensions** | **Baseline value** |
| --- | --- | --- | --- | --- |
| Cumulative OCV production and release | cumulative | doses | n/a | 0 |
| Doses shipped from supplier^27^ | dynamic | doses | n/a | 0 |
| DP batch process time | static | days | P | 2 |
| DP batch size | static | doses | P | 350,000 |
| DP capacity^28^ | dynamic | doses | P | n/a |
| DP labelling | static | days | P | 3 |
| DP maintenance switch^29^ | switch | dmnl | P | 0 |
| DP manufacturing decision^30^ | switch | dmnl | P | 1 |
| DP minimum campaign size | static | doses | P | 0 |
| DP orders^31^ | dynamic | doses | P | n/a |
| DP QA QC process time | static | days | P | 20 |
| DP visual inspection | static | days | P | 3 |
| DP facility set-up time | static | days | P | 0 |
| DS batch process time per serotype | static | days | P | 1 |
| DS batch size | static | doses | P | 350,000 |
| DS capacity^28^ | dynamic | doses | P | n/a |
| DS facility set-up time | static | days | P | 0 |
| DS line cleaning time | static | days | P | 3 |
| DS maintenance switch^29^ | switch | dmnl | P | 0 |
| DS manufacturing decision^30^ | dynamic | dmnl | P | 1 |
| DS minimum campaign size | static | doses | P | 0 |
| DS push orders^32^ | dynamic | doses | P | 0 |
| DS QA QC process time | static | days | P | 25 |
| DS waiting time per serotype | static | days | P | 2 |
| NRA release time | static | days | P | 28 (Shanchol, Euvichol), or 12 |
| Time to ship approved preventive orders | static | days | n/a | 7 |
| Time to ship approved reactive orders | static | days | n/a | 3 |
| Total WIP for DS^33^ | dynamic | dmnl | P | 0 |
| Total WIP for finished product^34^ | dynamic | dmnl | P | 0 |
| Transfer time to DP facility | static | days | n/a | 1 |

^27^ Determined based on the rate at which purchase orders are submitted from procurement agency to suppliers, following global policies on OCV use over time.

^28^ Determined based on the production queue and batch size. If pending production orders are larger than the batch size, then batch size scaled by the number of lines. The number of lines is determined based on the necessary facility capacity to meet annual production targets within one year, while keeping utilization high. If the pending production orders are smaller than the batch size, then capacity is equivalent to pending production orders.

^29^ Consider effect of planned facility maintenance, when production lines are idle: 0 – off, 1 – on. If on, user can specify period(s) of maintenance within the simulation.

^30^ Consider manufacturing of a given product: 0 – off, 1 – on

^31^ Equivalent to maximum DP capacity, pushes orders from the DS inventory to DP queue.

^32^ Equivalent to user-define production schedule. In this simulation, an annual production target is defined for each product, with production starting at the beginning of each year until target is reached.

^33^ Total inventory across the following stocks: DS queue, DS WIP, DS QA QC, and DS inventory

^34^ Total inventory across the following stocks: DP queue, DP WIP, DP QA QC, and DP lot release

*F.6 Market attractiveness*

Numerous factors impact supplier decisions to enter a vaccine market and subsequently stay or leave. In this model, three main dynamics are considered: 1) annual review of the OCV market size, 2) continuous review of excess inventory across all suppliers, and 3) change in countries priorities. The time to enter the market and scale-up capacity is defined by the user, while market attractiveness is assessed over the entire simulation period. Once a supplier exits a market, it is assumed that it will not come back. Each supplier has a different risk threshold for the minimum market size to stay in the market, considering both the previous year’s approved OCV requests and current pending decision letters. Additional, market saturation is assessed based on excess inventory across all suppliers. Arbitrary decisions to leave the market can be defined for each supplier at any time in the simulation. Parameter values can be found in Table 2 of the main text.

*F.7 OCV order fulfillment*

The public procurement process engages multiple stakeholders. UNICEF, in collaboration with GAVI and other partners, publishes public tenders inviting suppliers to make proposals. However, sporadic demand means it is difficult to estimate the exact volume that will be purchased during the tender period. Therefore, strategic demand scenarios are important to effectively communicate anticipated orders. They also help incentivize manufacturers to expand capacity, a time- and resource-intensive process, given the supply-constrained market. UNICEF signs long-term arrangements with selected suppliers and provides each a non-binding award (volume) over the tender period. This means that although suppliers with an award can expect to receive purchase orders, there is no guarantee as to the timing and volume of these orders. The most recently published tender covers the 2024-2028 period. In this subsystem, reactive orders are prioritized. Different decisions rules are defined for which purchase orders are fulfilled over time, depending on the available or expected inventory across suppliers.

**Table N:** Overview of model parameters – order fulfilment subsystem.

| **Parameter** | **Type** | **Units** | **Array dimensions** | **Baseline value** |
| --- | --- | --- | --- | --- |
| Average awarded price per dose | static | USD | n/a | 1.35 |
| Impact of suppliers on procurement prices^35^ | switch | dmnl | n/a | 0 |
| Effect of suppliers on procurement price^36^ | dynamic | dmnl | n/a | 0 |
| Operational costs for reactive OCV use per dose | static | USD | n/a | 0.65 |
| Operational costs for preventive OCV use per dose | static | USD | n/a | 0.65 |
| Ability to fulfil reactive orders^37^ | dynamic | dmnl | n/a | 0 |
| Ability to fulfil preventive orders^38^ | dynamic | dmnl | n/a | 0 |
| Impact of budget constrain on procurement^39^ | switch | dmnl | n/a | 0 |
| Overall budget allocated to OCV | static | USD | n/a | 275 million |

^35^ Consider effect the number of suppliers has on price competition: 0 – off, 1 – on.

^36^ Assume that beyond the first supplier, each new supplier will lead to a 10% decrease in the UNICEF awarded price, with a minimum of 1 USD. All suppliers receive the same UNICEF awarded price.

^37^ Logic for fulfilling reactive orders is based on available supply, unfulfilled decision letters for OCV use in reactive settings, and available budget.

^38^ Fulfilling preventive orders is only possible when pending orders for reactive use is zero. Logic for fulfilling preventive orders the depends on unfulfilled decision letters for OCV use in preventive settings and available budget.

^39^ Consider effect of budget constraint on procurement: 0 – off, 1 – on.

*F.8 Climate & conflict*

Many factors influence the spread of cholera in endemic countries, as well as the likelihood and magnitude of outbreaks. Extensive work has been done on better understanding social, environmental, and other drivers of cholera disease epidemiology. Environmental factors include rainfall and temperature, which can influence access to safe sources of water and spread of bacteria [5]. Additionally humanitarian crises such as armed conflict and forced displacements accentuate the risk of cholera, limiting access to reliable infrastructure and basic services [6]. High-level assumptions are made on the expected impact of climate and conflict on the risk of cholera transmission. These are considered to be relatively conservative estimates, although alternative values can be tested through sensitivity analysis.

**Table O**: Overview of model parameters – climate and conflict subsystem.

| **Parameter** | **Type** | **Units** | **Array dimensions** | **Equation** | **Baseline value** |
| --- | --- | --- | --- | --- | --- |
| Impact of climate dynamics on at risk population | switch | dmnl | n/a | n/a | 1 |
| Annual change in at risk population per country^40^ | static | dmnl | C | n/a | 2% |
| Impact of extreme climate and conflict dynamics on outbreaks | switch | dmnl | n/a | n/a | 1 |
| Annual change in outbreaks and  emergencies | static | dmnl | n/a | n/a | 3% |

^40^ Countries are classified into three relative risk levels the based on Notre Dame Global Adaptation Initiative’s vulnerability index [7]. A relative risk of 3 leads to a 2% annual increase in the country’s population at risk of cholera, while a relative risk of 2 and 1 leads to 1.5% and 1% increase, respectively.

**References**

1. Theil H. Applied economic forecasting. Amsterdam: North-Holland Pub. Co.; 1966.

2. UNICEF. Emergency stockpile availability - oral cholera vaccine. 2025 [cited 2 Apr 2025]. Available: https://www.unicef.org/supply/documents/emergency-stockpile-availability-oral-cholera-vaccine

3. GAVI. Vaccine Funding Guidelines (June 2024). In: Gavi, the Vaccine Alliance [Internet]. Jun 2024 [cited 1 Dec 2024]. Available: https://www.gavi.org/sites/default/files/support/guidelines-2024/GAVI-Vaccine-Funding-Guidelines-aug2024.pdf

4. Kim JH, Mogasale V, Burgess C, Wierzba TF. Impact of oral cholera vaccines in cholera-endemic countries: A mathematical modeling study. Vaccine. 2016;34: 2113–2120. doi:10.1016/j.vaccine.2016.03.004

5. Girotto CD, Behzadian K, Musah A, Chen AS, Djordjević S, Nichols G, et al. Analysis of environmental factors influencing endemic cholera risks in sub-Saharan Africa. Science of the Total Environment. 2024;926. doi:10.1016/j.scitotenv.2024.171896

6. Charnley GEC, Jean K, Kelman I, Gaythorpe KAM, Murray KA. Association between Conflict and Cholera in Nigeria and the Democratic Republic of the Congo. Emerg Infect Dis. 2022;28: 2472–2481. doi:10.3201/eid2812.212398

7. Chen C, Noble I, Hellmann J, Coffee J, Murillo M, Chawla N. University of Notre Dame Global Adaptation Initiative: Country Index Technical Report. 2024 Aug.

1. Link to ICG cholera vaccine dashboard: <https://www.who.int/groups/icg/cholera> [↑](#footnote-ref-2)
2. Link to GTFCC OCV dashboard: <https://apps.epicentre-msf.org/public/app/gtfcc> [↑](#footnote-ref-3)
