# Supplementary material for: Strategies for achieving a healthy oral cholera vaccine market: Model-enabled scenario exploration of supply and demand dynamics
Source: PLoS Negl Trop Dis. 2026 Feb 26;20(2):e0013995. doi: 10.1371/journal.pntd.0013995 (PMC12959686; doi:10.1371/journal.pntd.0013995)
Supplement: Attachment — Submitted filename: Ya To the editor Plos NTD.docx [file pntd.0013995.s002.docx]

Yaoundé, 9 Oct 2025.

Armand Nkwescheu

To the editor Plos NTD

Dear Sr,

I have reviewed the manuscript assigned to me.

I cannot submit my comments and recommendations because the toolbox does not display properly. It gives me only the opportunity to submit recommendation.

I accept the manuscript

I am worried as I have been trying for a couple of hours now.

Please, kindly advise.

Thanks
