# Supplementary material for: Strategies for achieving a healthy oral cholera vaccine market: Model-enabled scenario exploration of supply and demand dynamics
Source: PLoS Negl Trop Dis. 2026 Feb 26;20(2):e0013995. doi: 10.1371/journal.pntd.0013995 (PMC12959686; doi:10.1371/journal.pntd.0013995)
Supplement: Attachment — Submitted filename: Response to Reviewers.docx [file pntd.0013995.s004.docx]

26 January 2026

Dear Editors:

Thank you for taking the time to read our cover letter and consider the submitted manuscript (PNTD-D-25-01383, Strategies for achieving a healthy oral cholera vaccine market: Model-enabled scenario exploration of supply and demand dynamics). We are glad to read the positive review.

The authors would like to express gratitude to the editors and reviewers for constructive feedback and the opportunity to submit a revised version of the manuscript that addresses the points raised during the review process. Input from reviewers contributed to an improved piece. The manuscript continues to be timely given the continued shortage of cholera vaccines, despite persistent cholera outbreaks.

The following documents have been re-submitted for consideration: 1) this cover letter ('Response to Reviewers') with author responses to editor and reviewer comments, 2) an updated manuscript with track changes ('Revised Manuscript with Track Changes'), 3) an updated, clean manuscript ('Manuscript'), and 4) an updated, clean appendix (‘Appendix’) after correcting typos.

Additionally, several formatting updates have been made to better align with the journal’s requirements. For example, all main figures have been uploaded as separate Figure files in .tif format, with the correct size and resolution, as stipulated in the instructions provided.

We confirm that neither the manuscript nor any parts of its content are currently under consideration or published in another journal. All authors have approved the updated manuscript and agree with its submission to PLOS NTDs.

Sincerely,

Authors

| **Editor / Review comments** | **Author responses** |
| --- | --- |
| We have amended your Competing Interest statement to comply with journal style. We kindly ask that you double check the statement and let us know if anything is incorrect. | Thank you for the notice. We have reviewed and confirm the statement is correct. |
| Please ensure that the affiliations of the authors listed on the manuscript title page (CITY) do exactly match with the affiliations provided in the online submission form NOTE: Affiliations should include a department (if applicable), an institution, a CITY, and a country. | We confirm the affiliations on both the manuscript title page and online submission match. Our research center, being multi-disciplinary, does not typically include a department in author affiliations when publishing. The other elements are included – institution (KU Leuven), city (Leuven), and country (Belgium). We have removed the postal address, based on the structure provided for the affiliation. |
| We noticed that you used the phrase 'not shown' in the manuscript. We do not allow these references, as the PLOS data access policy requires that all data be either published with the manuscript or made available in a publicly accessible database. Please amend the supplementary material to include the referenced data or remove the references. | Thank you for the notice. We have removed the reference to ‘not shown’ as it does not change the reviewer’s or reader’s ability to understand the full meaning of the sentence. |
| Thank you for stating "Data supporting results can be found in the supplemental file or through public online sources referenced in the paper."  Please note that your Data Availability Statement is currently missing the repository name, and the DOI/accession number of each dataset OR a direct link to access each dataset. If your manuscript is accepted for publication, you will be asked to provide these details on a very short timeline. We therefore suggest that you provide this information now, though we will not hold up the peer review process if you are unable. | Thank you for the diligent feedback. We have reviewed the manuscript and supplementary information. The relevant data used for modeling is cited in the main text and appendix. All data come from published reports or peer-reviewed papers, except two sources which are online dashboards. We have added the relevant links to both in the statement. |
| Please provide an Author Summary. This should appear in your manuscript between the Abstract (if applicable) and the Introduction and should be 150-200 words long. The aim should be to make your findings accessible to a wide audience that includes both scientists and non-scientists. | A short author summary was provided in the initial submission, in the form of bullet points. This section has been adapted to better align with the journal’s requirements. The updated author summary can be found in **lines 84-102** of the clean, updated manuscript. |
| Your manuscript is missing the following sections: Methods. Please ensure all required sections are present and in the correct order. Make sure section heading levels are clearly indicated in the manuscript text, and limit sub-sections to 3 heading levels. | The relevant section has been renamed from ‘methodology’ to ‘methods’, to fit the journal’s criteria. Authors have checked that all required sections have been included in the manuscript. These changes can be found in **line 216** of the clean, updated manuscript. |
| We have noticed that you have uploaded Supporting Information files, but you have not included a list of legends. Please add a full list of legends for your Supporting Information files after the references list. | A list of figure and table legends appearing in the supporting information files have been added to the main manuscript file, after the references list. These additions can be found in **lines 945-1017** of the clean, updated manuscript. |
| Potential Copyright Issues:  i) Please confirm (a) that you are the photographer of 5A, or (b) provide written permission from the photographer to publish the photo(s) under our CC BY 4.0 license. | The image used in figure 5(a) is from a WHO website ([link](https://www.afro.who.int/news/who-secured-over-562-000-doses-oral-cholera-vaccine-pre-emptive-campaigns-south-sudan-2018)). A reference is included in the image (WHO, 2018). Regarding rights for using the image, we have found the following on WHO’s website: Permission from WHO is not required for the use of WHO materials issued under the Creative Commons Attribution-NonCommercial-ShareAlike 3.0 Intergovernmental Organization (CC BY-NC-SA 3.0 IGO) license. The  CC BY-NC-SA 3.0 IGO licence allows users to freely copy, reproduce, reprint, distribute, translate and adapt the work for non-commercial purposes.  However, since the image itself has no substantive value in the figure, the authors decided to adapt the figure since submitting the manuscript in August. An updated version of the interface uses a plain color (rather than image) background. This updated background is seen in a revised version of Figure 5(a). Therefore, no images are used, removing any copyright issues. |
| Kindly revise your competing statement in the online submission form to align with the journal's style guidelines: 'The authors declare that there are no competing interests.' | The competing statement in the online submission has been updated to align with the journal’s style guidelines. |
| The suitability of SD modeling is explained, but the specific reason for using SD over other modeling approaches is not justified. Please provide a comparative justification or approach that demonstrates its ability to capture feedback relationships and system-level policy effects. | The authors appreciate the suggestion to reflect further on the choice of SD over other modeling approaches. Beyond explaining its general suitability. In principle, other methodologies could be applied to analyze market dynamics for vaccines with unstable demand. However, they often lack the ability to represent the endogenous feedback relationships observed. On the other hand, SD was selected given its unique ability to effectively and transparently capture feedback dynamics that give rise to complex market behavior. In particular, the continuous time approach to SD modeling allows for accumulations and delays that reflect observed market dynamics. Since market dynamics (e.g., vaccination policies, procurement) are driven by aggregate feedback behavior rather than heterogeneous individual behavior, agent-based models were deemed to be less suitable. Furthermore, given the focus on policy exploration and multi-criteria evaluation of scenarios, optimization methods were deemed to be too narrow. While other methods can be useful to model one sub-system of the model (e.g., supply chain, procurement, etc.) SD is better suited to integrated dynamics across multiple domains and stakeholders. Reflections on the choice of SD over other modeling approaches can be found in **lines 221-233** of the clean, updated manuscript. |
| A long manuscript with many abbreviations, scenarios and assumption. But I would say i have just minor comments. Perhaps some fact-boxes - with scenario assumptions would make the manuscript more easily read and understood. | Thank you for the feedback. The authors have gone through the manuscript and made minor changes with the aim to further improve clarity. |
| First, this model considers the role of OCV, as well as complementary multisectoral interventions (as stated in the introduction), and your efforts and model is impressive. I understand that this comment may fall outside of your main scope, but I still miss an assumption of how many cases and/or deaths could be expected with the different scenarios – if that is possible. You call this later in the text (page19, line 552) significant reduction requested in reactive settings – and this could perhaps be translated to assumed number of prevented cases. If an increased cost is to be swallowed it would perhaps be important to show saved lifes and cases – even if that is not an easy task, and pit out how much closed to the goals set in the Global Task Force on Cholera Control one can come. You have commented something concering this issue in the discussion but it could perhaps be more highlighted in the abstract. | Thank you for the positive feedback. The authors appreciate the reflection on disease burden. The model focuses on market dynamics, using different KPIs for supply, demand, and order fulfillment – as defined in Figure 2. In this work, market health, determined by supply and demand aligning in quantity and over time, is a proxy for progress in advancing the objectives of the GTFCC around cholera control and by extension disease burden. Given complexity in assessing outbreak size, poor surveillance data, and exclusion of explicit disease transmission modeling (as described in the limitations), estimates for the impact of scenarios on disease burden are not provided. However, scenarios are expected to be better or worse for overall cholera control based on their ability to promote market health. This is clarified in a few instances in the results. The text in line 552 of the original manuscript has also been adapted to avoid confusion (see **lines 563-564** in the clean, updated manuscript). The author summary has also been adapted to demonstrate the use of market health as a proxy, see **lines 94-96**. |
| I am not totally confident that “phases” (tabl 3) are sufficiently explained for an uninformed reader. | Thank you for the feedback. A sentence has been added to add clarity on the meaning of phases within a preventive vaccine program. This change can be found in **lines 447-449** of the clean, updated manuscript. |
| It is good that assumptions into the model are referenced, but I can´t find why 3 further OCV will be available at 2028. Is it just an assumption or based on developer’s prognosis? | Thank you for raising this important point. The model assumes three new OCV suppliers will enter the market by 2030. As referenced in the text, **lines 403-405** of the clean, updated manuscript, this is based on publicly reported OCV development and technology transfer efforts. Also this was deemed as an appropriate assumption by stakeholders, as the market will likely be saturated with more than three new suppliers. This is reflected in some of the scenarios, where some suppliers leave the market after a few years due to decreased demand. |
| A small misspelling was discovered at page 12, line 369 concerning the timespan of the simulation period. | Thank you for flagging this typo. It has been adapted, as shown in **line 380** of the clean, updated manuscript. |
| At page 19 line 546 it is stated that 881 m doses are requested, but later that 75 m doses is sufficient. I guess that the first number refers to the whole simulation period. If that is correct please make clear. | Thank you for this comment. The sentence has been clarified to specify that the 881m is the total doses requested in scenario 1 between 2013 and 2025, as shown in **lines 559-560** of the clean, updated manuscript. |
